# Multivariate connectivity: A brief introduction and an open question

**DOI:** 10.3389/fnins.2022.1082120

**Published:** 2023-01-10

**Authors:** Mengting Fang, Craig Poskanzer, Stefano Anzellotti

**Affiliations:** ^1^University of Pennsylvania, Philadelphia, PA, United States; ^2^Columbia University, New York, NY, United States; ^3^Boston College, Chestnut Hill, MA, United States

**Keywords:** multivariate connectivity, pattern analysis, fMRI, neural networks, nonlinear interactions

## Introduction

Cognitive processes engage multiple interacting brain regions. To study these interactions, researchers analyze neural data with “connectivity” methods, that capture the temporal co-variation between the fluctuations in the responses at different locations in the brain. The responses at any given location can depend jointly on the responses at multiple other locations. Despite this, a vast majority of connectivity studies only focus on univariate interactions, either between one voxel and another, or between the average response in one region and the average response in another. By neglecting multivariate signals, univariate approaches to connectivity lead to inevitable information loss. In addition, to the extent that information is encoded by multivariate patterns of response, multivariate connectivity could provide more than just increased sensitivity, offering a qualitatively different understanding of the transformations of information between brain regions (Anzellotti and Coutanche, 2018; Basti et al., 2020).

In order to leverage multivariate information in the study of interactions between brain regions, several new techniques have emerged over the past decade. While in this article we focus on applications to fMRI, these techniques can be applied to a variety of data modalities (e.g., EEG, MEG, multi-site electrophysiology). Current techniques mainly differ in two aspects: (1) whether they are applied directly to BOLD responses or to derivative measures (e.g., decoding accuracy), and (2) how they model statistical dependence between regions. Some approaches are applied directly to BOLD responses. For example, a recent technique (Geerligs and Henson, 2016) estimates the statistical dependence between two regions by computing the multivariate distance correlation between their response patterns over time. This measure takes into account the BOLD signal across multiple voxels within each region, thus avoiding the information loss that would have occurred if those voxels had been averaged. Another technique uses transfer entropy (Lizier et al., 2011) as a measure of multivariate statistical dependence. Computing transfer entropy for high-dimensional patterns is computationally costly, therefore this technique is typically applied on small numbers of voxels or using approximations (see Bossomaier et al., 2016). Transfer entropy has the advantage that it can capture linear as well as nonlinear interactions. Another method that can capture both linear and nonlinear interactions—multivariate pattern dependence (MVPD) (Anzellotti et al., 2017; Fang et al., 2022)—trains a predictive model of the relationship between multivariate responses in different regions using part of the data (either directly or using principal components), and then tests the model's accuracy on the left-out data, using it as a measure of statistical dependence. Note that if a nonlinear relationship between two brain regions has a linear component, it can also be detected by linear methods.

Some techniques compute multivariate dependence and then apply additional analysis steps. Multivariate integration (Sasai et al., 2016) uses multivariate responses at a given timepoint to predict responses in future timepoints, and then compares the performance achieved by combining multiple predictor regions to that achieved with individual predictor regions in isolation to capture the interactions between multiple brain areas. Multi-Connection Pattern Analysis (MCPA, Li et al., 2017) characterizes the relationship between two brain regions during two different tasks using two separate models. Then, it tests whether the relationship is task-sensitive by comparing the model's predictive accuracy when trained during that same task than that during the other task.

Certain approaches compute the statistical dependence between measures derived from multivariate responses—such as the accuracy of a classifier. This strategy is adopted by informational connectivity (Coutanche and Thompson-Schill, 2013), which trains a multivariate classifier, and then tests the correlation between classification accuracies across multiple trials (or fluctuations in trials' similarity with prototypical patterns for the conditions). This offers the opportunity to evaluate multivariate dependence along specific dimensions of interest to the experimenter. Derivative measures are also used in representational connectivity (Kriegeskorte et al., 2008; Henriksson et al., 2015), which tests the similarity between representational dissimilarity matrices (RDMs) computed for different regions. A recent study extended this approach using Riemannian distance (Shahbazi et al., 2021), which can potentially capture nonlinear interactions. The features of different multivariate connectivity methods are summarized in Table 1. Comprehensive reviews of multivariate connectivity methods can be found elsewhere (Anzellotti and Coutanche, 2018; Basti et al., 2020), here we focus on selected examples to illustrate the variety of their possible applications.

**Table 1:** Summary of different multivariate connectivity methods.

**Method**	**Measurement of neural responses**	**Can be applied to resting state data**	**Captures nonlinearities**	**Predicts timeseries in independent data**
Multivariate distance correlation	Response patterns	Yes	Yes	No
Multivariate transfer entropy	Response patterns	Yes	Yes	No
MVPD	Response patterns	Yes	Yes (selecting a nonlinear mapping)	Yes
Multivariate integration	Response patterns	Yes	Potentially	No
MCPA	Response patterns	No	Yes	Yes
Informational connectivity	Decoding accuracy or correlations to mean responses	No	Potentially	No
Representational connectivity	RDMs	Yes	Potentially	No

Under the heading “Captures nonlinearities”, the entry “Potentially” indicates that the method can be extended in a straightforward manner to a version that captures nonlinearities.

## Examples of applications

Since multivariate connectivity methods have been introduced, they have yielded a variety of results across different areas of Cognitive Neuroscience. In one application, they have been used to show that representations of the same stimuli vary across different trials in a coordinated fashion across multiple brain regions (Henriksson et al., 2015). The authors compared RDMs across visual areas either during the same trial, or across different trials (Henriksson et al., 2015), and reported that RDMs were more similar when they were compared within the same trial, providing evidence for trial-by-trial fluctuations in the regions' response patterns.

Recent work studied what kinds of information transformations occur between brain regions. Basti et al. (2019) used multivariate linear models to predict response patterns in a target region based on response patterns in a predictor region, and computed three distinct metrics to characterize each interaction: goodness-of-fit, sparsity, and pattern deformation. Thanks to this approach, they observed that different dimensions of the responses in the predictor region are affected differentially (e.g., amplified or dampened) by the transformation that maps them onto responses in the target region.

Other studies used multivariate connectivity to investigate how information is combined across multiple brain regions. In a pioneering study, Coutanche and Thompson-Schill (2015) analyzed the convergence of shape and color information in the anterior temporal lobe (ATL). They showed participants images of visual noise and instructed them to look for objects varying in color and shape, and found that across trials, the accuracy of decoding objects from response patterns in ATL could be predicted by jointly analyzing the accuracy of decoding color from response patterns in area V4 and shape from patterns in lateral occipital cortex. A related approach—“feature specific informational connectivity”—has been used to investigate episodic memory representations (Bone et al., 2020).

More recently, Fang et al. (2019) used an extension of MVPD based on artificial neural networks to show that the angular gyrus is characterized by joint statistical dependence with multiple category-selective regions, suggesting that it might play a role in combining information about different kinds of objects. MVPD has also been used to study the multivariate dependence between fMRI responses across participants (Li et al., 2019).

Direct comparisons between multivariate and univariate connectivity methods indicate that multivariate methods are more sensitive (Coutanche and Thompson-Schill, 2013; Geerligs and Henson, 2016; Anzellotti et al., 2017). In addition, these methods have the potential to uncover not only whether but also how brain regions interact. In an elegant example (Basti et al., 2019), estimating explicitly a linear transformation matrix made it possible to determine not just the presence but the type of interactions between brain regions. These advantages come at a cost: the implementation of multivariate connectivity methods is more complex, hindering their broader adoption in the research community. To address this issue, a growing number of toolboxes have been recently developed.

## Available toolboxes

One of the first toolboxes—the Informational Connectivity Toolbox (https://lrdc.pitt.edu/coutanche/informationalconnectivity)—provides a collection of MATLAB scripts to run informational connectivity analysis. More recently, a toolbox for multidimensional connectivity implemented in MATLAB has been made available with the article “Multi-dimensional connectivity: a conceptual and mathematical review” (Basti et al., 2020). This toolbox (https://github.com/RikHenson/MultivarCon) has been developed to be flexibly applicable to both fMRI and EEG. Another recently introduced toolbox developed in Python, PyMVPD (Fang et al., 2022, https://github.com/sccnlab/PyMVPD), implements MVPD. This toolbox enables users to train multivariate models of the interactions between brain regions, and to test their accuracy on left-out data. PyMVPD offers linear regression models as well as artificial neural networks, and it is designed to enable users to customize their own models and evaluation metrics to suit specific research needs. Thanks to this functionality, PyMVPD makes it possible to compute linear as well as nonlinear multivariate statistical dependence.

When it comes to measures of transfer entropy, a fully multivariate approach that preserves the information in all voxels is computationally intractable. Various toolboxes help researchers compute multivariate transfer entropy with suitable approximation techniques. For example, the Java Information Dynamics Toolkit (JIDT) (Lizier, 2014) is a Google code project which provides open-source code for multiple information-theoretic measures. It offers classic information-theoretic measures as well as higher-level measures of information dynamics. MuTE (Montalto et al., 2014) is a MATLAB toolbox that implements three estimators of multivariate transfer entropy (i.e., linear estimator, binning estimator, nearest neighbor estimator) under either the classical uniform embedding or the non-uniform embedding. Finally, the Information Dynamics Toolkit (IDTxl) (Wollstadt et al., 2019; https://github.com/pwollstadt/IDTxl) is a Python toolbox that implements multivariate transfer entropy estimation for the effective inference of network dynamics.

## The puzzle of nonlinearity

As discussed in previous sections, an important way that multivariate techniques can offer novel insight into complex neural dynamics is by examining the type of interactions between brain regions, not just their presence. A key goal of connectivity is to study how information is transformed from brain region to brain region. A growing body of research has demonstrated that at a cellular level, information is nonlinearly transformed (Xu et al., 2012; Tran-Van-Minh et al., 2015; Gidon et al., 2020; Beniaguev et al., 2021; Lafourcade et al., 2022). For example, the input/output function of a single pyramidal neuron can be best approximated by a deep nonlinear neural network (Beniaguev et al., 2021). Nonlinearities are also observed at the level of local field potentials (Sotero et al., 2010; but see Ito et al., 2017). Therefore, it is important for connectivity methods to be able to capture nonlinear interactions between regions. As an additional argument, high-performing models of perception heavily rely on nonlinearities (Khaligh-Razavi and Kriegeskorte, 2014; Yamins et al., 2014; Balestriero and Baraniuk, 2018), suggesting that they are an essential part of neural computation. Therefore, if connectivity measures aim to elucidate the transformations of representations that underlie cognition, they need to also capture nonlinear statistical dependence. On these grounds, recent work has called into question whether linear models of neural activity are sufficient to fully understand the relationship between the activity in regions across the brain (Anzellotti et al., 2017): models of the transformation of information across the cortex should offer the flexibility needed to capture nonlinearities.

To avoid confusion, we need to note that even though the mapping between the spiking activity of neurons and the observed BOLD response is also nonlinear, here we focus instead on nonlinear relationships between the responses in one region and those in another region. Several methods have the potential to capture such nonlinear interactions, including transfer entropy, multivariate distance correlation, MVPD, and functional coordinates (Lizier et al., 2011; Geerligs and Henson, 2016; Anzellotti et al., 2017; Poskanzer and Anzellotti, 2022). Informational connectivity (Coutanche and Thompson-Schill, 2013), if paired with nonlinear decoding techniques, could also detect nonlinear interactions, and multivariate integration (Sasai et al., 2016) can also be extended naturally to capture nonlinearities. Nonlinear methods have the flexibility to capture a broader variety of interactions between regions (since typically they can also capture linear interactions), and the input-output relationships in individual neurons are better captured by nonlinear functions (e.g., Hodgkin-Huxley models for individual neurons, or sigmoid models for mean-field activation). Therefore, the most biologically plausible nonlinear models of neural interactions are likely to be more biologically plausible than the most biologically plausible linear models.

While linear models of connectivity are most commonly used to characterize regional interactions, several studies have reported evidence of significant nonlinear relationships between brain areas (Friston et al., 1994; Stephan et al., 2008; Marinazzo et al., 2011; Poskanzer et al., 2022). Despite nonlinear dynamics having been found across the brain, however, linear models remain popular due to sufficient performance and enhanced interpretability. To add to the interpretability of nonlinear models, we recently developed a method using a basis set of Hermite polynomials to estimate the functional relationship between brain regions (Poskanzer et al., 2022)—it is our hope that the ability to explicitly define the function that relates the activity between two brain regions will provide increased transparency to the future evidence of nonlinear cortical interactions.

Although there is a strong theoretical grounding for the study of nonlinear statistical dependence between brain regions, fMRI evidence is still limited (Cox and Savoy, 2003; Hlinka et al., 2011; Poskanzer et al., 2022). If nonlinear interactions should be widespread in theory, why are they difficult to identify? There are several obstacles to identifying these types of interregional interactions. For example, fMRI responses are subject to hemodynamic filtering (De Zwart et al., 2009), affecting the latency of the BOLD signal. In addition, fMRI signal is noisy—this noise could overshadow nonlinear dependencies. Finally, fMRI has limited temporal and spatial resolution. In particular, the averaging of the signal across thousands of neurons within each voxel can conceal the presence of nonlinearities. This problem is compounded by connectivity methods that investigate univariate dependence between brain regions by averaging across multiple voxels. Although multivariate methods cannot overcome the difficulties inherent to fMRI as an imaging technique, by using multivoxel patterns of response instead of averaging across voxels to obtain a univariate timecourse we can avoid compounding the spatial smoothing.

## Discussion

A growing body of multivariate and nonlinear methods for examining connectivity provide a promising starting point for future work. However, capturing nonlinear relationships between the representations encoded in different brain regions will require additional steps. First, noise could obfuscate such relationships. While popular denoising techniques have been shown to remove some spurious nonlinear interactions between brain areas (Poskanzer et al., 2022), improvements in denoising could reveal previously hidden nonlinear interactions. When applying denoising, it will be important to proceed with caution and to include control analyses to ensure that the denoising is not introducing artifactual nonlinear relationships. Second, nonlinear models tend to have more parameters than linear models. Hlinka et al. (2011) reported finding evidence of subtle nonlinear interactions when using large amounts of data; experiments including larger amounts of data for each participant (e.g., Allen et al., 2022) could help improve the parameter estimates, making it easier to detect nonlinearities. Finally, applying multivariate and nonlinear connectivity methods to data with higher spatial and temporal resolution, such as multi-site electrophysiology, could overcome the limitations due to the limited spatial and temporal resolution of fMRI. FMRI responses correlate with local field potentials and with spatial averages of single unit measurements (Issa et al., 2013), thus studying connectivity through multi-site single unit recordings (Bosman et al., 2012; Hart and Huk, 2020; Fernández-Ruiz et al., 2021) could potentially help to understand where the nonlinear interactions are lost in the steps from single unit recordings to fMRI, and to evaluate whether it is possible to recover them with adequate analyses.

Understanding the nonlinear transformation of information across the brain is a fundamental topic in the study of brain connectivity. By leveraging a wide array of multivariate analyses to study rich, high dimensional neural data, researchers can improve upon their ability to map the computational topography of the brain.

## Author contributions

MF wrote a draft of the following sections: “Introduction”, “Examples of applications”, and “Available toolboxes”. CP wrote a draft of the section “The puzzle of nonlinearity”. SA conceived the structure of the article and edited/supervised all sections. All authors contributed to the article and approved the submitted version.
